# Interbacterial competition mediated by the type VIIb secretion system

**DOI:** 10.1099/mic.0.001420

**Published:** 2023-12-20

**Authors:** Eleanor R. Boardman, Tracy Palmer, Felicity Alcock

**Affiliations:** ^1^​ Microbes in Health and Disease Theme, Newcastle University Biosciences Institute, Newcastle University, Newcastle upon Tyne, NE2 4HH, UK

**Keywords:** bacterial competition, contact-dependent inhibition, protein secretion, T7SS, toxin, type VII secretion system

## Abstract

Successful occupancy of a given niche requires the colonising bacteria to interact extensively with the biotic and abiotic environment, including other resident microbes. Bacteria have evolved a range of protein secretion machines for this purpose with eleven such systems identified to date. The type VIIb secretion system (T7SSb) is utilised by Bacillota to secrete a range of protein substrates, including antibacterial toxins targeting closely related strains, and the system as a whole has been implicated in a range of activities such as iron acquisition, intercellular signalling, host colonisation and virulence. This review covers the components and secretion mechanism of the T7SSb, the substrates of these systems and their roles in Gram-positive bacteria, with a focus on interbacterial competition.

## Introduction

The Sec and Tat machineries are general protein transport systems for export of proteins across the bacterial cytoplasmic membrane. The hallmark of Sec and Tat substrates is the presence of a well characterised N-terminal signal peptide of approximately 20–30 residues, which is required for targeting and translocation of these substrates. The defining feature of Gram-negative bacteria is the existence of an additional outer membrane, and over the years a number of specialised systems for secretion of proteins across the entire Gram-negative cell envelope have been identified and characterised [1]. These secretion systems function in a plethora of roles, including virulence and interbacterial antagonism. For a long time secretion systems were thought to be specific to Gram-negative bacteria; Gram-positive species lack an outer membrane and so transport of proteins by the Sec or Tat systems could result in extracellular localisation. An exception to this rule are the mycobacteria whose outer envelope contains a lipid bilayer which constitutes a non-canonical outer membrane [2].

The first clue to the existence of a specialised secretion system in Gram-positive bacteria came from the Actinomycetota with the identification of *Mycobacterium tuberculosis* ESAT-6, a small, secreted virulence factor with no identifiable N-terminal signal peptide to direct its secretion via either Sec or Tat [3, 4]. A family of ESAT-6-like proteins was found to be widely distributed in Gram-positive species. These have been designated WXG100 proteins, to account for their approximate 100 amino acid length and the presence of a conserved central Trp-X-Gly (WxG) motif (IPR010310). Genes encoding distant homologues of ESAT-6 were identified in species of Actinomycetota and Bacillota, often in the proximity of a gene encoding a membrane-bound FtsK/SpoIIIE-family ATPase (IPR002543), and based on these observations a Gram-positive type VII secretion system (T7SS) was proposed [5].

The *M. tuberculosis* T7SS was subsequently identified as a key virulence factor in mice [6–8], and its FtsK/SpoIII-family ATPase, EccC (esx conserved component C), was shown experimentally to be responsible for secretion of ESAT-6 and the related WXG100 protein CFP-10 [6, 8]. *M. tuberculosis* has since been found to encode five different secretion systems for ESAT-6 family proteins, named ESX-1 to ESX-5 [9], each with distinct functions (reviewed in [10]). A subsequent study in *Staphylococcus aureus* determined that the WXG100-family proteins EsxA and EsxB were secreted by an analogous system also requiring an FtsK/SpoIIIE family protein, named EssC, and these three proteins were shown to play a role in pathogenesis of murine abcesses [11]. Beyond the presence of WXG100- and FtsK/SpoIII-family proteins, the components of the T7SS in Actinomycetota are unrelated to those found in Bacillota, and the two systems have therefore been designated T7SSa and T7SSb, respectively. The T7SSb is widely distributed among pathogenic and non-pathogenic species of the Bacillota phylum, having been studied in species of *Staphylococcus, Streptococcus, Bacillus, Enterococcus* and *Listeria* [12–20].

## Components and assembly of the T7SSb

### Core components of the T7SSb

The WXG100 and EssC/EccC protein families are common to all T7SS. WXG100 family members are found almost exclusively in Actinomycetota and Bacillota, with a smaller number of sequences also found in Chloroflexota [21]. Intriguingly, a limited number of WXG100 sequences have also been found in certain Gram-negative species, alongside an FtsK/SpoIIIE-family protein, though these have not yet been confirmed functionally as secretion systems [22, 23].

**Fig. 1.**
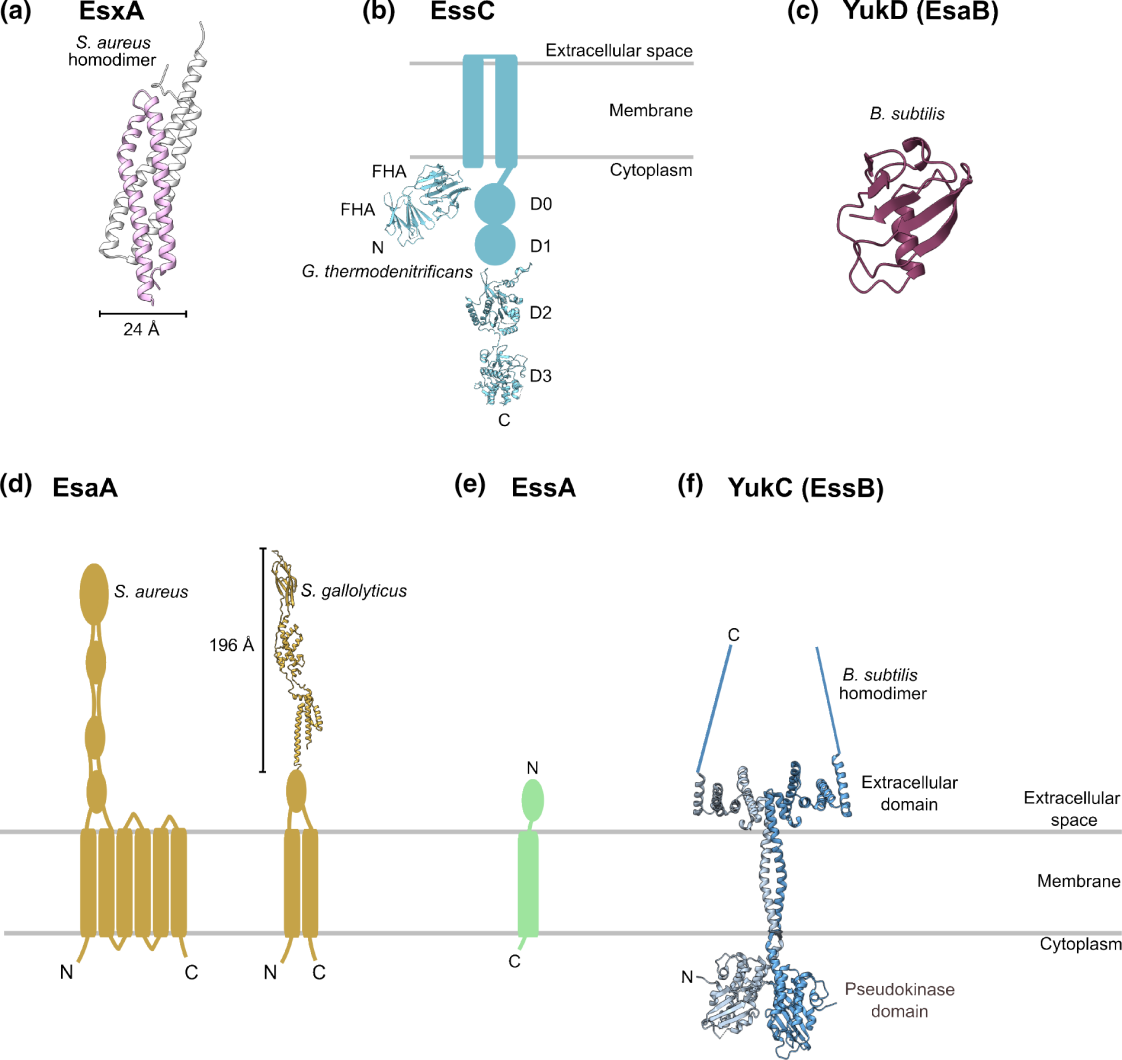
Structural arrangement of the T7SSb. (**a-f**) Schematic representations and experimentally-determined structures of core components of the T7SSb. (**a**) Crystal structure of the *S. aureus* EsxA homodimer (2VRZ) [26]. The two subunits (pink and white) assemble in a head-to-tail arrangement. (**b**) EssC encompasses two N-terminal forkhead-associated (FHA) domains, two transmembrane helices and four C-terminal ATPase domains, D0 – D3. Crystal structures have been determined for the N-terminal FHA domains (5FWH) and the C-terminal ATPase D2 and D3 domains (5FV0) from *G. thermodenitrificans* [28]. (**c**) Crystal structure of the *B. subtilis* EsaB homologue YukD (2BPS) [44]. (**d**) EsaA homologues have a variable number of transmembrane helices; shown here are the *S. aureus* and *S. gallolyticus* homologues. The crystal structure of part of the *S. gallolyticus* extracellular domain is shown (7JQE) [45]. (**e**) No structural information is available for EssA however its predicted topology shows a single transmembrane helix with N-terminal extracellular domain [51]. (**f**) Crystal structure of the *B. subtilis* EssB homologue YukC (6Z0F) [43]. The C-terminus is missing from the structure and is represented by a blue line.

The canonical T7SSa substrates ESAT-6 and CFP-10, encoded by the genes *esxA* and *esxB*, respectively, are WXG100 family members. Each has the helix-turn-helix structure and they are secreted as a folded heterodimeric four helix bundle [24]. The T7SSa membrane channel for secretion is formed by EccC, a membrane protein with four FtsK/SpoIIIE-family ATPase domains that is thought to couple ATP hydrolysis to substrate transport. The structure and function of T7SSa systems is reviewed in detail in [10, 25]. All T7SSb systems also encode and secrete a WXG100 protein, EsxA, which is distinguished from the mycobacterial subfamilies by forming a homodimer [26, 27]. The T7SSb EccC counterpart, EssC, is also a membrane protein containing four cytoplasmic ATPase domains, but differs from EccC by the additional presence of two N-terminal forkhead associated (FHA) domains [28]. Recent advances in structural analysis of the T7SSa have revealed how this >2 megadalton membrane complex is arranged, providing key insight into the mechanism of transport [29–31]. By contrast, current structural information on the T7SSb machinery is limited to a few individual components or domains, and a structural understanding of the T7SSb is still of utmost priority. Although T7SSb-encoding loci vary among Bacillota species six conserved components that constitute the core machinery are always encoded, and are each required for secretion [11, 32, 33]. Using the nomenclature established in *S. aureus *, these are EsxA, EsaA, EsaB, EssA, EssB and EssC. Current structural information available for T7SSb machinery components is summarised in Fig. 1.

### T7SSb components with T7SSa homologues: EsxA, EssC and EsaB

EsxA is a small helical hairpin of the WXG100 family which homodimerises prior to secretion [12, 26] (Fig. 1a), in contrast to the heterodimeric T7SSa WXG100 substrates. *esxA* is typically encoded upstream of the other T7SSb components and is in some cases transcribed separately from the rest of the locus, as seen in group B *Streptococcus* (GBS) and some strains of *S. aureus *. In other strains of *S. aureus* as well as *S. gallolyticus *, *esxA* is co-transcribed with the rest of the T7SS structural genes, although RNAseq data show that *S. aureus esxA* transcript levels are much greater than for other T7SS genes [15, 33–35]. Some species carry two copies of *esxA*, the significance of which is not yet known [16, 36]. Although frequently described as a T7SS substrate, EsxA is also required for secretion of all substrates so far tested, though its role in secretion is yet to be defined.

**Fig. 2.**
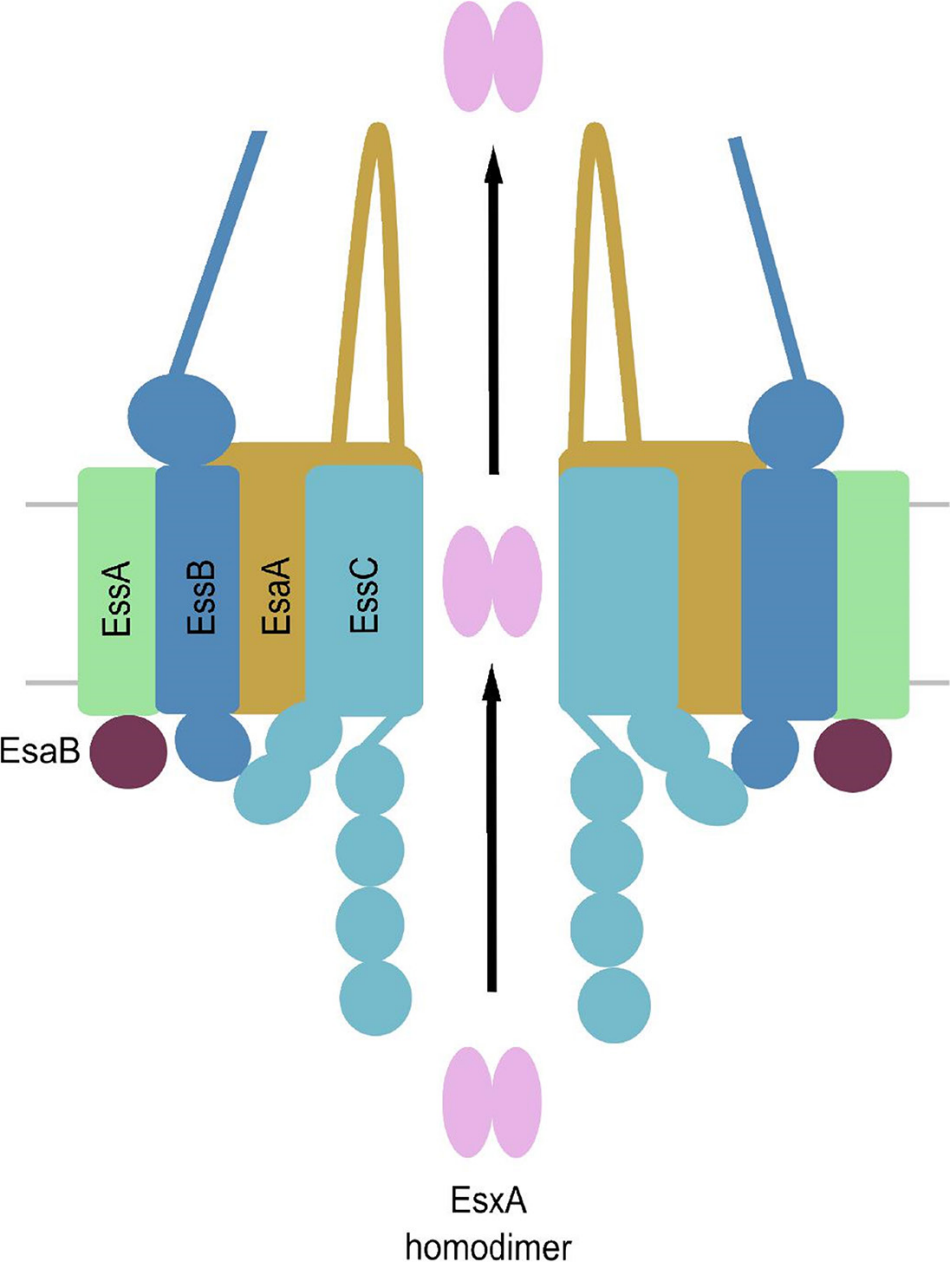
Model for the structural organisation of the T7SSb. A model for the assembled T7SSb, based on T7SSa architecture and known T7SSb intersubunit interactions. EssC FHA domains are required for association of EssA and EssB, but not EsaA. EsaA and EssC are thought to interact via their transmembrane domains and bacterial two hybrid studies suggest that EssA might recruit EsaB to the translocon [43].

The central component of the T7SSb membrane channel is EssC (Fig. 2). EssC contains two transmembrane helices and assembles as a hexamer [28–30]. The membrane domain is flanked by two cytoplasmic domains: an N-terminal region containing two FHA domains, and a C-terminal region containing four ATPase domains (D0, D1, D2 and D3) (Fig. 1b). The only structural data available for EssC is for individual fragments of the soluble domains [1–4], however the likely channel structure can be inferred from structures of the T7SSa translocon, since the EccC membrane and ATPase domains are conserved in EssC. It is therefore anticipated that the six copies of EssC transmembrane helix two line the transmembrane channel. Conformational changes in the ATPase domains are proposed to accompany regulated opening and closing of the secretion channel [30]. The central channel width is unclear as published structures of intact T7SSa complexes likely represent closed conformations at less than 10 Å. However based on homology modelling of EssC with the hexameric DNA translocon FtsK this channel is predicted to open to 30 Å, a dimension that would be wide enough to accommodate a folded dimer of WXG100 proteins [12, 21, 26, 28, 30].

The four EssC ATPase domains comprise a large cytoplasmic extension (Fig. 1b) which functions in substrate recognition and is additionally thought to power secretion via ATP hydrolysis [28, 31, 37–39]. This region of EccC is flexible beyond the D0 domain and it has been proposed that the ATPase domains adopt different arrangements within the cytoplasm which could reflect the activation status of the complex [29]. FHA domains, as found at the EssC N-terminus, contain phosphothreonine recognition motifs which mediate signal transduction in a variety of cellular pathways. However, the FHA domains of EssC are lacking the conserved phosphate-binding residues of other bacterial FHA domains [40] suggesting a novel function independent of phosphothreonine signal transduction. MxiG is a transmembrane subunit of the *Shigella flexneri* Type III secretion translocon inner membrane ring complex, and facilitates assembly of the cytoplasmic sorting platform via interaction with the cytoplasmic subunit MxiK. Interestingly MxiG, like EssC, harbours an FHA fold but lacks phosphothreonine binding activity [41], hence these domains might mediate similar functions. Indeed recent copurification studies support a role for the EssC FHA domains in inter-subunit interactions, specifically with the pseudokinase domain of EssB (see below) [42, 43]. A second soluble core component of the T7SSb is EsaB, an ~11 kDa cytoplasmic protein with a ubiquitin-like fold (IPR024962) [44] (Fig. 1c). The same fold is found in the N-terminal cytoplasmic domain of the much larger (~54 kDa) T7SSa component EccD, that scaffolds other T7SSa membrane components [30, 31]. Preliminary studies with the *B. subtilis* EsaB homologue YukD support the idea that EsaB might, like EccD, interact with other transmembrane membrane components, specifically EssA and/or EssB [43].

### T7SSb-specific core components: EsaA, EssA and EssB

The second largest T7SSb subunit after EssC is EsaA. EsaA contains between two and six transmembrane helices depending on the species, but in common is a large extracellular portion, the structure of which has been solved by two groups independently. It forms an elongated dimer that is long enough to span the thick peptidoglycan cell wall (Fig. 1d) [38, 45]. This is in agreement with previous observations that the *B. subtilis* and *S. aureus* EsaA homologues are surface-exposed and the former can function as a phage receptor [46–48]. As the only T7SSb component with the ability to span the cell wall, EsaA has been proposed as a conduit for export of effectors but direct experimental evidence of this is currently lacking.

Two smaller membrane proteins, EssA and EssB, are also conserved across T7SSb systems. EssA comprises a single transmembrane helix and an extracellular domain, and very little is known about its role in secretion [33]. EssB, which also has a single transmembrane helix, has an N-terminal cytoplasmic pseudokinase-like domain and a C-terminal extracellular domain through which the protein dimerises. The extracellular domain is hypothesised to interact with other components, substrates or possibly the cell wall [43, 49]. The pseudokinase domain of EssB, while lacking catalytic residues, retains structural similarity to the substrate-binding region of protein kinases [49]. This domain has been purified in complex with the EssC FHA domains indicating that EssC and EssB interact at least partly through interaction of these domains [43].

### Translocon assembly and isolation

Attempts to isolate an intact T7SSb membrane complex have met with limited success to date, and structural data on translocon architecture are lacking. Co-purification experiments with tagged components suggests the existence of a constitutively-assembled translocon in *S. aureus* strain USA300 with interactions detected between all six core components [42, 50], and many of these interactions have been similarly detected in *B. subtilis* [43]. However, an alternative study failed to detect interactions between membrane components in *S. aureus* RN6390 [51] indicating that translocon assembly might be dynamic, and its regulation might be strain-specific. A preliminary model for translocon architecture is presented in Fig. 2 based on the available data for inter-subunit interactions, which are summarised in Table 1.

**Table 1.** Summary of interaction studies for T7SSb core components. Experiments were undertaken in *S. aureus* or *B. subtilis *. The *S. aureus* nomenclature is used throughout the table. BACTH refers to the bacterial adenylate cyclase two-hybrid system.

Protein(s)	Interaction details	Reference
EssC	Hexamerisation (also based on EccC studies).	[28–30]
EssC, EssB, EsaA, EssA	Co-purification of the four membrane proteins with affinity tagged EssB or EssC, from *S. aureus* membranes.	[42, 50]
EssC, EssB	Direct interaction between the EssC FHA domain and EssB pseudokinase domain, by BACTH and copurification of recombinant proteins from *E. coli*.	[43]
EssC, EssB	Co-purification of EssB with affinity tagged EssC from *S. aureus* membranes. Independent of EsaA but requires the EssC FHA_2_ domain.	[42]
EssC, EssA	Co-purification of EssA with affinity tagged EssC from *S. aureus* membranes. Requires the EssC FHA_2_ domain, EssB and EsaA.	[42]
EssC, EsaA	Co-purification of EsaA with affinity tagged EssC from *S. aureus* membranes. Independent of the EssC FHA_2_ domain.	[42]
EsaA, EssB	EsaA interaction with EssB transmembrane domain, by BACTH.	[43, 120]
EssA, EssB	EssA – EssB direct interaction by BACTH.	[43]
EssA, EsaB	EsaB – EssA direct interaction by BACTH.	[43]

### Genetic variability among T7SSb loci

**Fig. 3.**
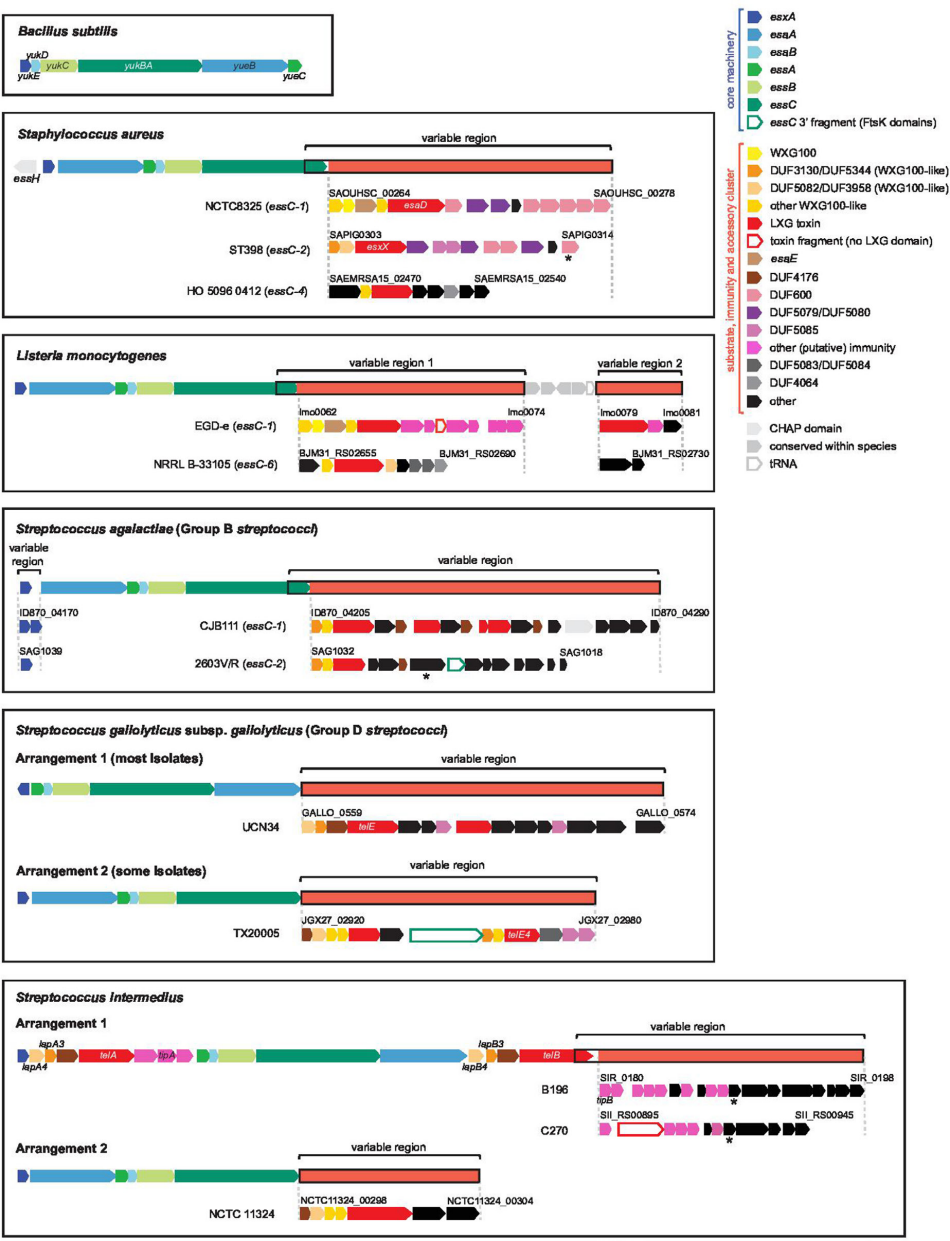
T7SSb genetic organisation in different organisms. The genetic organisation of T7SSb loci is shown for selected species. For each species the overall organisation of the T7SS locus is shown at the top with conserved core components in blue/green and substrate and accessory clusters in red. The substrate and accessory clusters are highly variable within species, and in some species the 3′ end of *essC* varies with this downstream region. Genetic arrangements within the variable regions are shown for selected strains, with the *essC* variant for that strain given in brackets where applicable. The example arrangements shown are non-exhaustive, and diagrams are not to scale. Genes with frequently occurring conserved domains are colour coded. Some domains were not identified by Interpro but were classified by HHPred search. *S. agalactiae* T7SS loci can carry between zero and two copies of *esxA*, and *esxA* in *S. gallolyticus* (arrangement one) is on the opposite strand. * Denotes a pseudogene.

Although their genetic arrangement can vary, the genes encoding the core T7SSb components are well conserved and are usually encoded at the 5′ end of T7SSb loci. By contrast, the 3′ ends of T7SSb loci exhibit a high level of genetic diversity, both between and within species [15, 20, 35] (Fig. 3). This variable downstream region encodes strain-specific substrates, accessory secretion factors, immunity genes and proteins with as yet unidentified functions. The sequence at the 3′ end of *essC*, encompassing the final two ATPase domains (D2 and D3), can also vary with the downstream gene repertoire, reflecting the role of these domains in substrate specificity and recognition [38, 42, 52].

Although highly divergent in sequence, these variable regions of T7SSb loci encode similar types of genes across different species, including members of the WXG100 and LXG domain families, which are the two major families of T7SSb substrate identified to date. In addition to genes encoding LXG and WXG100 domains, genes encoding particular ‘domains of unknown function’ including DUF5082, DUF3130, DUF4176, DUF600 are frequently observed across T7SSb loci (Fig. 3). These likely encode additional components, substrates or immunity proteins and the functions of several of these are beginning to be elucidated (discussed below). While many substrates and secretion factors are encoded at T7SSb loci directly downstream of the core machinery, T7-associated genes are also found at additional loci in some organisms. For example, the *S. aureus* LXG-substrate TspA, and the *S. agalactiae* T7-associated WXG100 protein SAG0230 are both encoded away from the T7SSb locus [36, 53].

A further feature specific to *S. aureus* T7SSb loci is the *essH* gene that is encoded divergently from *esxA* in all *S. aureus* isolates (Fig. 3). EssH is a CHAP domain-containing peptidoglycan hydrolase required for T7SSb substrate secretion in *S. aureus* [54]. This gene is not usually found at other T7SSb loci and may be a specific adaptation to the *S. aureus* cell envelope, although a gene encoding a CHAP domain-containing protein is present at the *S. agalacitae* CJB111 T7SSb locus, so this domain is not unique to *S. aureus* T7 systems (Fig. 3).

Unusually, the *B. subtilis* T7SSb locus is represented by a six gene cluster (*yukEBCD-yueBC*) encoding only the core secretion components, and lacks genes for LXG substrates or accessory factors at the cluster [13]. However, genes for up to seven distinct strain-specific LXG toxin-immunity pairs can be found at different loci throughout the chromosome [55], and typical T7SSb-associated genes (e.g. DUF5082-encoding) are also found at some of these loci.

## The T7SSb as a bacterial weapon

### Variable contribution of the T7SSb to virulence in pathogenic strains

Mycobacterial T7SSa systems have diverse roles, for example in nutrient acquisition and horizontal gene transfer [10]. The role of the ESX-1 system in infection and pathogenesis has been studied in depth, and the WXG100 family substrates ESAT-6 and CFP-10 are important virulence factors secreted by ESX-1 [6–8]. *S. aureus* EsxA and EsxB are both WXG100 proteins that have been implicated in virulence. A strain lacking any of *esxA*, *esxB* and *essC* was attenuated in a murine abscess virulence model [56], and deletion of the whole *S. aureus* T7SSb locus attenuated both virulence and nasal colonisation in a murine cystic fibrosis model [33] although this attenuation was only observed for two of three *S. aureus* strains tested. Another study found *S. aureus esxA* and *esxB* to have a role in modulating apoptosis during infection of human epithelial cells [57].

In the GBS organism *S. agalactiae* an *esxA* mutant was attenuated in a murine hematogenous meningitis virulence model and EsxA was found to contribute to cytoxicity in brain endothelial cells [16]. The same study observed a pore-forming activity for EsxA in synthetic bilayers. Pore-forming activity has also been reported for *M. tuberculosis* ESAT-6 and was speculated to promote cytotoxicity and virulence, however this was subsequently shown to be artifactual and the lysis of host membranes by ESX-1 occurs via an alternative, unknown mechanism [58]. The ESAT-6 homologues EsxE and EsxF have since been reported to insert into and destabilise membranes *in vitro* [59], however the *in vitro* pore forming activity of both *S. agalctiae* EsxA and *M. tuberculosis* EsxE-EsxF is highly dependent on pH, and in both cases the physiological relevance has yet to be determined.

Efficient colonisation is a prerequisite for virulence, and diverse infection models have been used to examine a potential role for the T7SSb in host colonisation. For example, the T7SS was shown to be important for murine vaginal tract colonisation by *E. faecalis* [60], and for colonisation of the murine gut by *S. gallolyticus* [34], with the latter study also demonstrating a specific function of the T7SS in adherence to human colorectal cancer cells. By contrast, the T7SS was not involved in colonisation of the murine nasopharynx by the mouse-adapted *S. aureus* WU1 strain [61]. In a murine model of *L. monocytogenes* infection, colonisation of the spleen and liver were unexpectedly impaired by T7SS-dependent secretion of EsxA [62]. Furthermore, a study using a murine model of female genital tract colonisation to compare T7SSb subtypes across different strains of GBS (* Streptococcus agalactiae *) found the T7-dependence of host colonisation to vary across strains [35]. Taken together these data suggest that the strain-specific T7 substrate repertoire may determine host phenotypes in colonisation and virulence studies.

While the T7SSb contribution to pathogenesis has been documented in several studies, not all T7SSb-carrying species are pathogenic and alternative functions in iron acquisition and cell signalling have been proposed [63, 64]. An investigation of the *Bacillus velezensis* T7SS in colonisation of Arabidopsis roots demonstrated that secreted YukE (the *Bacillus* EsxA homologue) caused leakage of iron from plant root cells for uptake by the bacterium, thus promoting colonisation [65].

### T7SSb-secreted toxins mediate interbacterial competition

**Fig. 4.**
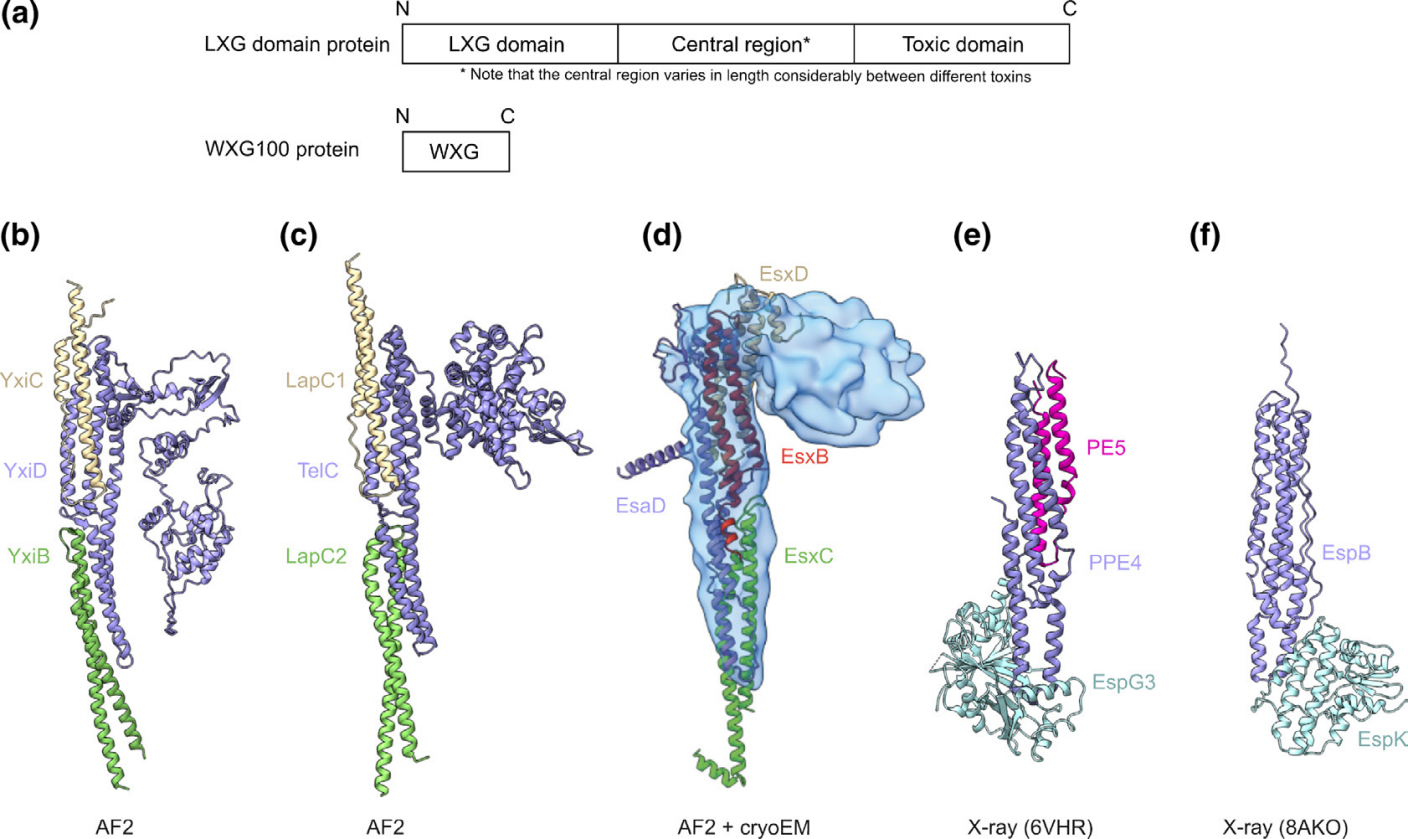
Structures of T7SS substrates. (**a**) Schematic showing the domain organisation of LXG toxins and WXG100 proteins. (b–d) AlphaFold models of T7SSb substrate complexes (* B. subtilis* YxiD, *S. intermedius* TelC and *S. aureus* EsaD). In each model the LXG protein is coloured lilac, and the WXG100-like partners green, yellow and red. In (**c**) the EsaD sequence used for the prediction was truncated at 200 amino acids to remove the C-terminal domain which cannot be confidently predicted. The low-resolution volume of the EsaD-EsxB-EsxC-EsxD-EsaE-EsaG hexamer, determined by cryo-EM [98], is overlaid providing experimental support for the model. (e–f) Experimentally determined structures for two T7SSa substrates, in complex with their targeting chaperones (* M. tuberculosis* PE5-PPE4-EspG3 [116], and *M. tuberculosis* EspB-EspK [119]). All structures are oriented with the export arm at the top.

In addition to the functions discussed above, accumulating evidence over the last decade points to a major role for the T7SSb in bacterial antagonism. Proteomic and genomic studies have identified polymorphic antibacterial toxins as a diverse and ubiquitous class of T7SSb substrate, distinct from but related to the WXG100 proteins [5, 18, 53, 66–68]. These proteins are large, multidomain toxins which typically have an N-terminal α-helical domain, a central region of variable length, and an extremely polymorphic C-terminal toxin domain (Fig. 4a).

Such proteins were first identified bioinformatically due to the similarity between the α-helical N-terminus and the WXG100 family [5, 18]. The helical N-terminal domains have been bioinformatically categorised as either ESAT-6-like (IPR010310) or, more commonly, LXG domains (IPR006829), and all belong to the WXG100 superfamily [66]. Genes encoding LXG proteins are widely distributed across the Bacillota phylum, and are particularly prevalent in classes Bacilliales, Lactobacilliales and Clostridiales [66, 67]. As outlined above, they are frequently encoded downstream of the core T7SSb components (Fig. 3), but can also be found at additional loci [18, 53, 55]. Comprehensive analysis of *Listeria monocytogenes* genomes has identified fourteen chromosomal hotspots where LXG proteins can be encoded away from the T7SSb locus [20, 69].

The similarity of LXG domains to known WXG100 T7SS substrates, and the frequent association of LXG toxins with T7SSb loci suggests a role for the T7SSb in interbacterial competition via toxin secretion. A study of the *S. aureus* nuclease toxin EsaD was the first to demonstrate this experimentally, showing that *S. aureus* strains overproducing EsaD were able to kill related strains lacking the EsaD immunity protein, EsaG [70]. A detailed study of *Streptococcus intermedius* identified three T7-secreted LXG toxins, TelA, TelB and TelC. TelB and TelC were tested in competition assays and found to inhibit strains of related bacteria [67]. Subsequent studies have identified a wide variety of additional LXG toxins with predicted antibacterial properties, some of which have been shown experimentally to mediate T7-dependent antagonism in *S. aureus, E. faecalis* and *B. subtilis* [53, 55, 71].

The term LXG was introduced on account of a conserved (L/F)xG sequence motif within these proteins [66, 67]. LXG domains are specific to the T7SSb and target the protein to the secretion apparatus [72]. No functions have yet been ascribed to the central regions of these proteins, which are not conserved, are of variable length, and are sometimes annotated as a ‘PT’ (pre-toxin) domain. Some toxins such as *S. intermedius* TelC and *B. subtilis* YxiD lack this central region and comparative analysis may therefore provide clues to its function(s). The toxin domain at the C-terminus is the most variable, with 56 types having been identified in strains of *L. monocytogenes* alone [20, 69]. They include predicted membrane-depolarising toxins, nucleases, NADases, lipid II phosphatases and ADP ribosyl transferases as well as a number of domains of unknown function. Unexpectedly, a recent report identified a new class of LXG toxin where the modular structure is reversed with the toxin domain located at the protein’s N-terminus. The targeting domain is not simply appended to the C-terminus of the protein, but the entire sequence is reversed and so carries a GxL motif. These proteins, named TslA (Type VII secreted lipase A), are distributed widely among Bacillota and are also verified T7SSb substrates [73].

Since LXG toxins are antibacterial an immunity protein is essential to protect the producing cell and its kin. This is usually achieved by binding to and neutralising the toxin, and the immunity gene is frequently encoded adjacent to its cognate toxin. LXG toxins are polymorphic not only in the type of toxin they carry, but toxins of the same family found in related strains also exhibit sequence variability. Reflecting this variability, immunity genes are often found in tandem arrays of multiple non-identical copies, with variable ability to bind and neutralise the toxin made by that particular strain [70, 74].

Protective immunity gene clusters are also found in potential target strains which lack the cognate toxin proteins [66, 75]. These are frequently found in alternative strains of the same species, indicating that T7SSb-mediated competition targets highly similar strains. Furthermore, non-kin strains of the same species, which are likely to be highly competitive, frequently carry dissimilar toxin-immunity systems (Fig. 3), and all experimentally tested T7SSb are capable of intraspecies competition [55, 67, 70, 71, 76, 77]. Potential interspecies Gram-positive prey have been identified for the *S. gallolyticus* T7SS (* S. pyogenes* and *E. faecalis* are both susceptible) and the *E. faecalis* T7SS (* E. faecium *, *S. aureus* and *L. monocytogenes* are all susceptible). However not all Gram-positive species are susceptible, and of several tested Gram-negative organisms, none were susceptible to inhibition by *S. intermedius *, *E. faecalis *, or *S. suis* [67, 71, 77].

### Toxic activities of LXG substrates

TspA is an LXG toxin found across all *S. aureus* strains as well as other *Staphylococci*, *Listeria* and *Enterococci*, and is encoded adjacently to its cognate immunity protein TsaI [53]. *S. aureus* TspA is encoded away from the T7SSb locus and multiple *tsaI* homologues are encoded at the same locus. Following secretion, TspA is thought to remain associated with the producing cell, although some of the protein may also be released into the environment. TspA acts as a bacteriostatic toxin through depolarisation of membranes by forming ion channels, rather than large nonselective pores, and is active against other *S. aureus* strains when tested in a zebrafish larval model of bacterial competition [76]. The unusual, reverse-substrate TslA is also carried by *S. aureus* and like TspA, is encoded distal to the T7SSb locus. TslA carries a C-terminal GXL targeting domain, and the N-terminal toxic domain shows phospholipase A activity. It is neutralised though tight interaction with its cognate immunity protein, TilA [73].

Additional *S. aureus* toxins are encoded at the T7SSb locus and are specific to the *essC* variant carried by that strain. *essC1* variants encode EsaD, whose C-terminal endonuclease domain degrades the chromosomal DNA of competitor *S. aureus* [70]. Immunity to EsaD is provided by the product of an immediately downstream gene, EsaG. Recently a crystal structure of this toxin-immunity protein complex has shown an unusual rearrangement of the EsaD nuclease domain upon immunity protein binding, where EsaG sandwiches itself between two halves of the EsaD nuclease to destabilise and unfold the active site of the protein [78]. EsaG is a DUF600 family member and DUF600 proteins are found widely in strains of *S. aureus,* where some strains will encode multiple copies of variant DUF600s [15, 79]. More broadly, EsaG is a member of the SUKH superfamily of nuclease immunity proteins, whose toxins are extruded by multiple types of secretion system [66]. Toxins with DNase activity are common T7SSb substrates [66, 80]. *B. subtilis* strain NCIB3610 encodes six LXG toxins on its chromosome, five of which are predicted or known DNases and include the *yeeF-yezG* module that resembles *esaD-esaG* from *S. aureus* [55, 70, 81]. *essC2* strains of *S. aureus* do not possess EsaD but instead encode EsxX, a potential pore-forming toxin [68], and *essC3* and *essC4* strains carry alternative LXG proteins downstream of *essC* which have yet to be biochemically characterised (Fig. 3).

*Streptococcus intermedius* LXG domain proteins include TelA, TelB, TelC and TelD [67]. TelA toxicity has been demonstrated via expression of the toxin domain in the *E. coli* cytoplasm, although currently the nature of TelA toxicity is unknown as neither the protein nor its immunity protein TipA bear homology to characterised proteins [67]. The TelB and TelD toxins are also active intracellularly. TelB is an NADase, and although the activity of TelD is yet to be designated it is thought to be a membrane-depolarising toxin, similar to TspA, since their immunity proteins belong to the same family [67, 82]. TelC is a lipid II phosphatase that cleaves peptidoglycan precursors [67]. Although lipid II exists on both sides of the cytoplasmic membrane, TelC is active specifically at the outer face of the cell membrane. The active enzyme requires a bound calcium ion which is likely to bind extracellularly, accounting for its lack of toxicity in the cytoplasm. Its immunity protein TipC has a single transmembrane segment and an extracellular-facing TelC-inhibitory domain, consistent with the outer membrane surface being TelC’s site of action [74]. While cytoplasmically expressed TelC is non-toxic, curiously T7SS-secreted TelC is also non-toxic to either the producing cell or neighbouring cells in liquid cultures. TelC toxicity has been demonstrated in two situations – either when the toxin domain alone is artificially re-routed for export via the Sec translocon by addition of a signal peptide, or when the full-length protein is secreted by the T7SS during growth on a solid surface, allowing for cell-cell contact [67, 74]. These results indicate that to exert its toxicity TelC must be delivered specifically to the inner cell wall, either directly by the producing cell via the Sec translocon, or alternatively from the T7SS of a neighbouring cell in a contact-dependent manner. It therefore follows that unlike the Sec translocon, the T7SS delivers TelC from the cytoplasm to the extracellular milieu, traversing the entire cell envelope in a single step, and the peptidoglycan cell wall forms a barrier preventing toxins like TelC from accessing their target at the cell membrane.

A further TelE LXG toxin has also been described in *Streptococcus gallolyticus* [17]. TelE carries a bacteriocin-like glycine zipper motif, which inserts into and destabilises target membranes, and is homologous to *S. aureus* EsxX.

### Contact-dependence of T7SSb-mediated antagonism

The use of toxins in intercellular competition is widespread among prokaryotes, and a variety of toxin delivery mechanisms have evolved. Whereas some toxins, e.g. bacteriocins, are released into the environment and encounter target cells via diffusion, others require cell-cell contact to exert their effect. The experimental evidence to date points to a contact-dependent mechanism for T7SSb-dependent bacterial antagonism. Contact dependence was demonstrated for both *S. intermedius* and *E. faecalis* T7SSs, and separating attacker and prey strains with a porous membrane was found to prevent T7SS-mediated inhibition. Moreover, purified toxins or toxin-containing culture supernatants had no activity when applied directly to prey cells [67, 71]. A study of *B. subtilis* found each of the six chromosomally-encoded LXG toxins in strain NCIB3610 to be active in competition, but only during biofilm growth, again suggestive of contact-dependence [55].

The fundamental mechanisms for contact-dependent inhibition in established systems vary significantly. The proteobacterial CDI (contact-dependent inhibition) system (reviewed in [83]) utilises a Type Vb secretion system with just two specialised proteins, CdiAB, required to deliver toxic effector proteins to prey cells of the same species or family [84]. The outer membrane-localised CdiB translocates the toxin-carrying CdiA protein from the periplasm to the cell surface, where the β-helical CdiA N-terminus forms a surface-attached filament. Upon contact with a prey cell, CdiA binds to a prey outer membrane protein. Delivery of the toxin, often a nuclease, into the target cell cytoplasm depends on hijacking of endogenous prey outer and inner membrane proteins as receptors, by domains within CdiA. By contrast the type VI secretion system (T6SS), also found in Gram-negative species, consists of a large contractile sheathed tube that spans the inner and outer membranes of the cell. The T6SS uses mechanical force to inject toxins into target cells in a receptor-independent manner. T6SSs can target a wide range of organisms including bacteria, archaea, fungi and mammalian cells [85, 86], this diversity probably facilitated in part by the lack of requirement for a receptor on the target cell.

There is a lack of evidence for the existence of a T7SSb tube or needle-like structure, however EsaA has been proposed as a possible candidate for binding to target cells, due to its surface-exposure outside the cell wall and its lectin-like tip [38]. T7SSb toxins need to traverse either the peptidoglycan cell wall or the entire cell envelope of the prey cell to reach their site of action, and the evidence suggests that factors belonging to the aggressor cell will be essential for this process [67, 71].

### Regulation of T7SSb activity

Secretion systems are large macromolecular machines whose synthesis and assembly is tightly regulated. In the case of the T7SSb this has hampered investigation of its function since it frequently has low or no activity in typical laboratory conditions of planktonic monoculture [33, 87]. In *S. aureus* and *L. monocytogenes *, T7SSb upregulation has been linked to infection and colonisation [62, 88, 89], and while a number of pathways have now been implicated in T7SS regulation the overall picture is unclear, not least due to significant intra- as well as inter-species differences in regulation.

In *S. aureus *, the global transcription factor SigB plays a key role in regulating the T7SSb, and negatively regulates *esxA* transcription [90], likely via a complex regulatory circuit involving the ArlRS two-component system, the Agr quorum sensing system, and DNA-binding proteins SpoVG and SarA [90–93]. Additional SigB-independent factors such as the SaeRS two-component system also regulate T7SSb gene expression [94, 95]. Different *S. aureus* strains frequently have mutations in these pathways, providing one explanation for observed differences in secretion activity [33, 63, 94, 96–98]. In terms of external stimuli, a reduction in membrane fluidity, achieved either by a decrease in temperature or incorporation of externally added cis-unsaturated fatty acids, has been found to stimulate *S. aureus* T7SS expression [99]. *In vivo* this is proposed to correspond to the lower temperature found in the host nasal cavity, frequently colonised by *S. aureus *, and/or the incorporation of host fatty acids from serum and nasal secretions, both of which increase membrane rigidity. Post-translational processes also regulate *S. aureus* T7SS activity: flotillin scaffold proteins found in membrane microdomains (similar to lipid rafts) have been observed to promote T7SSb assembly [100] and hemin post-translationally stimulates T7SS activity in certain strains [101].

In *B. subtilis* transcription of the T7SS structural genes is controlled by the DegSU two-component system. DegSU also controls biofilm development and sporulation, and therefore the expression of *B. subtilis* genes is activated in biofilms [87, 102]. Similarly, expression of *B. subtilis* LXG toxin-immunity pairs requires growth on solid medium in biofilm-promoting conditions, however here the regulation varies since expression of only a subset of these is dependent on DegSU. T7-dependent competition in *B. subtilis* modulates biofilm morphology and the spatial segregation of two strains within a biofilm, and a specific role for the YFJ toxin, encoded by *yfjB* and *yfjC*, has been proposed in biofilm-specific intercellular signalling, where toxin secretion modulates DegSU activity in neighbouring cells creating a feedback loop [55, 64].

Toxin secretion by other systems is frequently activated in response to attack from a nearby aggressor, as has been observed for both colicins and the T6SS [103–105]. A similar response has been demonstrated in *E. faecalis *, where phage infection of a T7+ strain was shown to stimulate T7SS-dependent killing of a T7-, phage-resistant prey strain, apparently via secretion of a single LXG toxin [71]. Sub-inhibitory concentrations of the membrane-disrupting antibiotic daptomycin were shown to mediate a similar effect indicating that membrane rupture by the phage might be the T7-activating event. Both phage infection and treatment with daptomycin were shown to increase T7SS gene expression, which is activated by a GntR-family transcription factor in an IreK-dependent manner [19, 71]. IreK is a transmembrane PASTA (penicillin-binding and serine/threonine kinase associated) domain-containing Ser/Thr kinase, involved in cell wall homeostasis, and is predominantly found in Bacillota and Actinomycetota. Signalling networks downstream of IreK are complex; it has multiple downstream phosphorylation targets, and functions in both conserved and species-specific pathways [106, 107]. The IreK-mediated response to phage- or daptomycin-induced membrane damage in *E. faecalis* is highly specific, since the T7SS was not activated by treatment with membrane-destabilising bile salts, nor by the cell wall-inhibiting antibiotic ceftriaxone, which also activates an IreK-dependent stress response [71]. Interestingly the DNA-damaging antibiotic mitomycin C also activated T7SSb transcription leading to speculation that T7 upregulation might be activated in response to T7 attack from both membrane-permeabilising and nuclease toxins, activities frequently associated with LXG domains [108]. Collectively, data suggest that stressors stimulating T7 activity are specific, and pathways can vary between strains, perhaps reflecting the specific environmental challenges faced by different strains.

## Strain-specific secretion factors and T7SS targeting signals

### Small helix-turn-helix proteins as export factors

While the core T7SSb apparatus is well conserved across species, the number and type of substrates is highly variable across species and strains, and there is growing evidence that different substrates require substrate-specific export factors. WXG100 proteins have low sequence homology and are defined by a helix-turn-helix fold with a WxG motif in the turn. Genes encoding small WXG100-like proteins are frequently found in T7^+^ organisms, often in the neighbourhood of LXG genes (Fig. 3). WXG100-like proteins lack the WxG motif, but like WXG100 proteins are also ~100 residues long and have a helical hairpin fold, and can be classified in one of a number of DUF families (Fig. 3).

The function of these proteins has been investigated in *S. intermedius *, where the *telC* and *telD* LXG toxin genes are immediately preceded by a pair of WXG100-like genes, designated Lap (LXG-associated α-helical protein) and named *lapC1-lapC2* or *lapD1-lapD2*, respectively. Lap1 is a DUF3130 protein and Lap2 a DUF3958 protein. They form a stable pre-secretion complex with the LXG domain of their cognate toxin, and are critical for its secretion [67, 82]. The *telA* and *telB* genes of the same organism are similarly preceded by genes encoding a pair of small helical proteins, which belong to the DUF5344 and DUF5082 families, designated Lap3 and Lap4, respectively. Here the genetic organisation is slightly different with a third protein encoded between the WXG100-like pair and the toxin (Fig. 3). Lap3 and Lap4 form a stable complex with their cognate TelA/B toxin, and so are predicted to function analogously to Lap1 and Lap2 [67, 72], leading to speculation that all LXG proteins might require a pair of WXG100-like proteins. In agreement with this hypothesis it was recently reported that three small WXG100-like proteins are required for secretion of the *S. aureus* LXG toxin EsaD [109]. EsxB is a bona fide WXG100 protein, whereas EsxC and EsxD have a predicted WXG100-like fold but lack the canonical WXG motif. All three proteins function as EsaD-specific export factors, binding stably to the EsaD LXG domain and directing its secretion [109], analogously to the Lap1-Lap2–LXG presecretion complexes of *S. intermedius. esxB*, *esxC* and *esxD* are located in a four-gene cluster along with *esaE*, directly upstream of *esaD* (Fig. 3), a synteny which is conserved among *esaD*/*yeeF* homologues. Similarly, secretion of the reverse substrate TslA also utilises a WXG100-like pair of export factors, Tla1 and Tla2, which are encoded immediately downstream of the toxin, and likely bind analogously to its reverse-LXG domain [73].

*S. aureus* EsxC, EsxB and EsxD have previously been shown to be secreted substrates, suggesting they are co-secreted with EsaD. Similarly, two small WXG100-like proteins encoded directly upstream of the *S. gallolyticus* LXG toxin TelE are T7-secreted [17]. The streptococcal Lap proteins are secreted with their cognate LXG domain protein in some cases but not all [72, 82], although it remains a possibility that these proteins are degraded following secretion and so not detected in the secretome. Why EsaD-like proteins reqiure three, rather than two, helix-turn-helix export factors is unclear, but might reflect a structural divergence of its LXG domain.

### T7SS targeting signals

T7SS substrates lack classical cleavable N-terminal signal peptides. While no equivalent universal signal sequence has yet been described, a number of conserved motifs have been identified and implicated in targeting of T7SS substrates. Early work on the T7SSa identified the seven C-terminal residues of CFP-10 as being required for its secretion. These residues are not conserved but form an amphipathic helix which binds specifically to a hydrophobic pocket on the D3 ATPase domain of the cognate EccC protein [24, 37]. Thirteen amino acids before its C-terminus, CFP-10 harbours a conserved YxxxD/E motif which is also required for secretion of the ESAT-6/CFP-10 dimer [110]. Surprisingly, the same YxxxD/E motif can be found on each of three unrelated classes of T7SSa substrate (CFP-10 family proteins, PE proteins and EspB/EspC) and in each case is required for secretion, suggesting it is a general ESX secretion signal found on all types of substrate [110]. This YxxxD/E motif was subsequently found to be part of an *H*xxxD/Exx*h*xxx*H* consensus motif (where ‘*H*’ stands for highly conserved hydrophobic and ‘*h*’ for less conserved hydrophobic residue) in a comprehensive alignment of 680 WXG100 proteins, including ESAT-6-like proteins, CFP-10-like proteins and homodimeric EsxA homologues of T7SSb systems [21]. The signature residues form a continuous surface along one face of an α-helical segment which protrudes from the WXG100 dimer, dubbed the export arm, and is spatially adjacent to the WxG motif of the other protomer.

A study of the *B. subtilis* EsxA homologue YukE demonstrated that truncation of the C-terminal export arm by even one amino acid severely compromises YukE secretion, and so like CFP-10, the extreme C-terminus contributes to targeting and secretion. Moreover, the same study demonstrated that the WxG motif is also critical for secretion, and so proposed these two regions to comprise a bipartite, spatially continuous secretion signal [12]. Indeed, in the crystal structure of the *M. tuberculosis* EspB substrate the tyrosine residue from the export arm’s YxxxD motif interacts directly with the tryptophan of the adjacent WxG motif [111]. However, a direct role for the WxG motif in binding to the secretion machinery has yet to be established.

The conservation of WxG and YxxxD/E-like secretion signals across different types of T7SSa substrates, as well as T7SSb EsxA family substrates suggests the same targeting mechanism exists across all T7 substrates. This prediction has been confirmed for the *S. intermedius* LXG toxin TelD, whose LXG domain forms a complex with the small WXG100-like export factors LapD1 and LapD2. The C-terminus of LapD1 constitutes the export arm for this complex, carrying a conserved FxxxD motif which is critical for TelD secretion [82]. Similarly, the leucine residue of the TelA LxG motif is also required for secretion [72]. LXG presecretion complexes adopt a conserved architecture with conserved residues on the Lap protein’s export arm located adjacent to the toxin’s LxG motif, suggesting these two motifs function as a bipartite secretion signal for LXG toxin presecretion complexes, analogous to that found in WXG100 dimers [72]. These commonalities will inform future investigation into targeting determinants for all classes of T7SSa and T7SSb substrates, their interactions with the secretion machinery and how targeting specificity is achieved.

### Structural similarities between T7SSa and T7SSb substrate complexes

Perhaps unsurprisingly in light of their conserved, bipartite secretion signals, T7SSa and T7SSb substrates share conserved structural features despite a lack of sequence homology. As previously discussed, WXG100 protein substrates are secreted as a dimeric, four-helix bundles in both T7SSa and T7SSb secretion systems. Two additional classes of T7SSa substrate – EspB, and the PE-PPE dimeric substrates – have been structurally characterised and both form helical bundles ~110 Å long and 20–30 Å (or four helices) wide (Fig. 4) [112–114]. There are no LXG proteins associated with the T7SSa, however members of the PE and PPE protein families can also be fused to highly variable C-terminal domains [115].

The recent structural characterisation of T7SSb LXG substrate complexes has revealed a striking similarity to structurally characterised T7SSa substrates. Cryo-EM analysis of the EsaD presecretion complex (a heterohexamer of the EsaD toxin, the small helical partners EsxBCD, the targeting chaperone EsaE and immunity protein EsaG) showed a cane- or tomahawk-like structure with a long helical shaft and a flexible head domain. Although only low-resolution volumes were determined, comparison with AlphaFold predictions suggest that the helical shaft comprises EsaD_LXG_-EsxBCD and has a cross-sectional diameter of 25–30 Å, consistent with the predicted width of the EssC channel (Fig. 4d) [109]. These findings are in agreement with the recently reported high resolution crystal structure of a TelA_LXG_-LapA3-LapA4 complex, with a similar helical rod conformation of ~180 Å × ~30 Å [72]. Both of these structures are consistent with AlphaFold predictions and bear a striking resemblance to structurally characterised T7SSa substrates, such as EspB or PE5-PPE4 (Fig. 4b-f) [111, 114, 116]. These observations suggest that the helical shaft of any substrate protein with the correct conformation and dimensions could be threaded through the secretion channel following its sequence-specific docking at the EssC/EccC ATPase domain.

### Globular chaperones as targeting factors

*M. tuberculosis* encodes five separate T7SSa systems, named ESX-1 – ESX-5, and 168 PE-PPE protein pairs, each of which must be targeted to the correct ESX system. On their own, the secretion signals described above are not sufficient for targeting and specificity. Each ESX system also encodes an EspG family chaperone which acts as an adapter, binding to the substrate protein and delivering it to the correct secretion system [110, 113, 117]. Several crystal structures detail how EspG binds to one end of the substrate helical shaft (Fig. 4e) [113, 114, 116, 118]. In addition to its small helix-turn-helix partner proteins, the *S. aureus* EsaD presecretion complex contains a globular chaperone, EsaE, which is encoded within the conserved *esaD* gene cluster. EsaE binds to an N-terminal region of EsaD encompassing both the LXG domain and the central region of unknown function. EsaE targets the presecretion complex to EssC, and is required for efficient EsaD secretion [70, 109]. The AlphaFold-predicted structure for the EsaE chaperone shows a remarkably similar fold to the EspG family globular chaperones utilised by the T7SSa and is therefore hypothesised to bind to the tip of the EsaD presecretion complex helical shaft and perform a mechanistically equivalent role to EspG [109]. Interestingly, *B. subtilis* YfjC, which is encoded immediately downstream of a T7-associated predicted pore-forming toxin YfjB, also shares predicted structural homology with EspG, despite having no sequence similarity to either EspG or EsaE proteins [64].

Some T7SSa substrates utilise an alternative chaperone unrelated to EspG. The *M. tuberculosis* substrate EspB shares structural similarity but not sequence homology with the PE-PPE family. EspB does not depend on EspG for targeting, but binds to the structurally distinct chaperone EspK [119]. The EspB-EspK complex is reminiscent of PPE-EspG complexes, with the globular chaperone domain bound to the tip of the EspB helical bundle (Fig. 4f), suggesting EspK might perform an EspG/EsaE-like targeting function, and a conserved fold is not essential for this role.

Finally, while *esaE* is specific to *esaD* loci, other LXG-protein interacting factors may exist among the proteins of unknown function encoded within T7SSb locus variable regions. For example DUF4176 proteins are frequently encoded at T7SSb substrate clusters (Fig. 3) and secretion of the *S. intermedius* TelA toxin has been shown to depend on the DUF4176 protein encoded immediately upstream [72].

### Concluding remarks

The T7SSb field has progressed considerably in recent years. Biochemical studies of membrane complex assembly pave the way for further structural studies, and the discovery of conserved targeting signals and structural homology across the broad range of all known T7SSa and T7SSb substrates will accelerate determination of the secretion mechanism. However, substrate export through the EccC/EssC channel is just the first step, beyond which the T7SSa and T7SSb are likely to diverge significantly due to the very different cell envelopes of the two groups of organisms. In the context of T7SSb-mediated interbacterial competition important questions to be addressed include how substrates traverse the producer cell wall, how toxins reach and gain access to the target cell, and how secretion is regulated in response to the presence of competitors. In the context of virulence, careful studies will be required to unpick the specific roles of T7SSb effectors in host interactions from the secondary effect of attenuated virulence resulting from impaired colonisation by less competitive T7-deficient strains. The potential additional roles of toxins beyond competition, in cell signalling and modulating cooperative behaviour within communities, is another compelling avenue for future research.
